# Bone Mechanical Properties and Mineral Density in Response to Cessation of Jumping Exercise and Honey Supplementation in Young Female Rats

**DOI:** 10.1155/2015/938782

**Published:** 2015-06-15

**Authors:** Somayeh Sadat Tavafzadeh, Foong Kiew Ooi, Chee Keong Chen, Siti Amrah Sulaiman, Leong Kim Hung

**Affiliations:** ^1^Sports Science Unit, Universiti Sains Malaysia, 16150 Kubang Kerian, Kelantan, Malaysia; ^2^Pharmacology Department, Universiti Sains Malaysia, 16150 Kubang Kerian, Kelantan, Malaysia; ^3^Musculoskeletal Research Laboratory, Department of Orthopaedics and Traumatology, The Chinese University of Hong Kong, Shatin, New Territories, Hong Kong

## Abstract

This study investigated effects of cessation of exercise and honey supplementation on bone properties in young female rats. Eighty-four 12-week-old Sprague-Dawley female rats were divided into 7 groups: 16S, 16J, 16H, 16JH, 8J8S, 8H8S, and 8JH8S (8 = 8 weeks, 16 = 16 weeks, S = sedentary without honey supplementation, H = honey supplementation, and J = jumping exercise). Jumping exercise consisted of 40 jumps/day for 5 days/week. Honey was given to the rats at a dosage of 1 g/kg body weight/rat/day via force feeding for 7 days/week. Jumping exercise and honey supplementation were terminated for 8 weeks in 8J8S, 8H8S, and 8JH8S groups. After 8 weeks of cessation of exercise and honey supplementation, tibial energy, proximal total bone density, midshaft cortical moment of inertia, and cortical area were significantly higher in 8JH8S as compared to 16S. Continuous sixteen weeks of combined jumping and honey resulted in significant greater tibial maximum force, energy, proximal total bone density, proximal trabecular bone density, midshaft cortical bone density, cortical area, and midshaft cortical moment of inertia in 16JH as compared to 16S. These findings showed that the beneficial effects of 8 weeks of combined exercise and honey supplementation still can be observed after 8 weeks of the cessation and exercise and supplementation.

## 1. Introduction

Since osteoporosis has become one of the major public health problems in men as well as women and with the cost of treatment rising every year, strategies to prevent this disease and lower the risk of related fractures are warranted. Early physical activities along with adequate nutritional intake have been prescribed for maximizing bone mass and minimizing bone loss in later life. Throughout the growing years, skeleton is most responsive to exercise. Therefore, physical activity during adolescence and young age could be one of the most significant determinations of peak bone mass [1, 2]. As reported in numerous studies, mechanical loading applied by exercise can result in increases in bone mass and strength [3–6]. However, various types of exercises have different stimulatory effects on bone, and particularly those weight-bearing exercises that generate high impact load on the skeleton are associated with greater skeletal responses [6–10] as compared to non-weight-bearing exercises [1, 11].

Besides physical activity, nutrition is another lifestyle factor that influences bone health. The positive effects of different nutritional supplements such as soy or soy isoflavones, calcium, and vitamin D supplementation alone on bone have been reported [12–14], when they were combined with physical activity [15, 16]. Honey which has been used as a food preserving agent for a long time is also well known for its beneficial actions in treatment of various medical diseases [17, 18]. Honey has been reported to increase calcium absorption after acute feeding in growing rats [19], implying that it may have potential in enhancing bone health.

Although the effects of increased mechanical loading on skeleton are obvious, the skeleton's ability to preserve the exercise-induced bone gain after the cessation of exercise is still equivocal. Some animal studies reported the preservation of exercise benefits [8, 20], whereas others have shown the loss of the exercise-induced bone gain after the termination of exercise [21, 22]. Since the effects of combined exercise and honey supplementation and their cessation on bone properties have not been investigated, the present study was conducted to evaluate the effects of cessation of jumping exercise and honey supplementation on tibia bone properties in young female rats.

## 2. Materials and Method

### 2.1. Animals

Eighty-four eleven-week-old Sprague-Dawley female rats were obtained from Laboratory Animal Research Unit, Universiti Sains Malaysia, and were placed in the experimental room. After one week of acclimatization to the environment, the rats were weighed for their initial body weight. Then the rats were block-randomized into seven initial body-mass-matched groups with 12 rats per group (6 rats per cage). Temperature and humidity of the room were maintained throughout the study under constant temperature of 24°C and relative humidity of 70–75%. The rats were exposed to a constant 12 : 12 light/dark cycle, with the light period starting from 7.00 p.m. to 7.00 a.m. for the entire experimental period. The reversed light/dark cycle was implemented to allow jump training during the day. At the end of the experimental period, the rats were weighed once again in order to obtain the final body weight. They were then anaesthetised, one at a time, by placing them for 2-3 minutes in a desiccated jar containing a chloroform soaked gauzed pad, before being decapitated using a small guillotine (Scientific Research Instrument, UK). The left hind leg was dissected for the measurement of bone properties. The research protocol was approved by Animal Ethics Committee of Universiti Sains Malaysia (AECUSM), number: USM/Animal Ethics Approval/2008/(39)(121).

### 2.2. Animal's Grouping

In this study, the rats with initial body mass of 190–220 grams were randomly assigned to seven groups, with twelve rats in each group (*n* = 12): 16 weeks of sedentary condition without supplementation group (16S), 16 weeks of jumping exercise group (16J), 16 weeks of honey supplementation group (16H), 16 weeks of combined jumping exercise and honey supplementation group (16JH), 8 weeks of jumping exercise followed by 8 weeks of sedentary group (8J8S), 8 weeks of honey supplementation followed by 8 weeks of sedentary group (8H8S), and 8 weeks of combined jumping exercise and honey supplementation followed by 8 weeks of sedentary group (8JH8S). One rat from 16J group was excluded due to skin infection. The rest of the rats were sacrificed at the age of twenty-eight weeks.

### 2.3. Training Programme

Rats in the exercise groups were trained to jump using a previously described protocol [8, 9]. Each rat was placed in a specially designed wooden box, measuring 30.5 × 30.5 × 40 cm in length, width, and height, respectively, and with a copper strip base that formed an electrical grid. The jumping exercise was initiated by applying electrical stimulation to the base of the box through a stimulator (Grass S48, USA). The stimulator was set to automatically deliver a stimulus of 50–80 V for 1 second and at 3-second intervals. To begin the exercise session, the rats were placed on the electrical grip with the stimulator turned off. When the stimulator was turned on, the rats jumped from the floor of the box to catch the top edge of the box with their forepaws. Upon reaching the top, they were then immediately repositioned by hand to the floor of the box to repeat the procedure. Jumping exercises were carried out from 8:30 a.m. to 11:30 a.m. Each rat was subjected to the exercise for a duration of 5 minutes per day for 5 days per week. The requirement for electrical stimulus decreased over time when the rats became accustomed to the jumping exercise.

The jump training began with an initial jumping height of 20 cm, after which the height was increased gradually to 40 cm by the third day. The rats that refused to jump were stimulated by the low voltage of electrical stimulation. The sedentary rats in the control group (free cages activity) were not given any electrical stimulus. In order to mimic the stress induced by handling before and after jumping exercise, the sedentary rats were handled 5 days per week for 16 weeks.

### 2.4. Honey Supplementation

Rats in honey group and combined jumping and honey group received honey as oral supplementation at the dosage of 1 g/kg body mass/rat/day via force feeding (gavages), 30 min prior to the jumping exercise [23]. Body mass of the rats was measured biweekly and the dosage of honey was calculated based on the most recent body mass.

### 2.5. Cessation and Continuous Jumping Exercise and Honey Supplementation

Jumping exercise was terminated after 8 weeks of exercise in 8J8S group. At the same time, no honey supplementation was given to the rats in 8H8S group after 8 weeks of honey supplementation. The rats in 8JH8S group underwent sedentary life without jumping exercise and honey supplementation after 8 weeks of combined exercise and honey supplementation. During the 8 weeks of cessation phase, the rats maintained their normal cage activity and handling was performed by the researcher for the rats 5 days per week. Rats in 16J, 16H, and 16JH groups had continuous exercise, honey supplementation, or combined exercise and honey supplementation regimen, respectively, for the whole experimental period, that is, 16 weeks.

### 2.6. Bone Harvesting and Measurements

After sacrificing the rats, the left hind tibiae of the rats were dissected. After removal of the flesh from the tibiae, the tibiae were then soaked in saline to prevent dehydration. The tibiae were then put into labelled plastic bags and stored at −80°C (Heto Ultra Freezer 3410, Denmark) for the subsequent measurements.

### 2.7. Micro-CT Analysis of Bone Mineral Density, Cortical Area, and Midshaft Cortical Moment of Inertia (MOI)

On the night before the measurement of bone mineral density and geometry, bones were thawed by keeping them at 4°C. On the day of bone densitometry measurement, the bones were placed in a custom-made plastic container designed for rats' tibiae. The proximal regions of the bones were then scanned using microcomputed tomography (micro-CT) scanner (XtremeCT, Scano Medical, Bruttisellen, Switzerland) with a resolution of 40 *μ*m, and a total of 100 slides were selected to contour and then were subsequently calculated using the built-in XtremeCT software programme for the measurement of bone mineral density and geometry parameters including tibial proximal total bone density, tibial proximal trabecular bone density, tibial midshaft cortical bone density, tibial cortical area, and tibial midshaft cortical moment of inertia [24].

### 2.8. Tibia Bone Mechanical Properties Testing

Before mechanical testing, the tibiae were taken out of the freezer and thawed overnight at a cold room temperature of 4°C. The mechanical properties of the bones were examined at midshaft of each bone using a three-point bending test by a material test machine (H25KS; Hounsfield Test Equipment, UK). A 25 N load cell was used to test the bones until failure to withstand this pressure. The tibiae were positioned horizontally and the distance between the two bottom supports of the tester was set 16 mm apart, with the cross-head speed set at 10 mm·min^−1^. After positioning each bone on the support, load was constantly applied and was directed vertically to midshaft. The tibial maximum force and tibial energy at the time of breaking were recorded.

### 2.9. Statistical Analysis

Statistical Package for Social Sciences (SPSS) version 19.0 was used for the statistical analysis. All the data are presented as mean ± standard deviation (SD). One-way analysis of variance (ANOVA) was performed to determine the significant differences between groups. When the one-way ANOVA revealed a significant difference,* post hoc* test (least significant differences test) was used to determine the differences between specific means. A “*p*” of <0.05 was considered statistically significant and used for all the comparisons.

## 3. Results

Initial body weight of the rats did not significantly differ among the groups. After sixteen weeks of experiment, no significant changes in body weight of the rats were observed between the groups (Table 1).

### 3.1. Tibial Total Bone Mineral Density (tBMD), Trabecular Bone Mineral Density (trbBMD), and Midshaft Cortical Bone Mineral Density (crtBMD)

**Figure 1:**
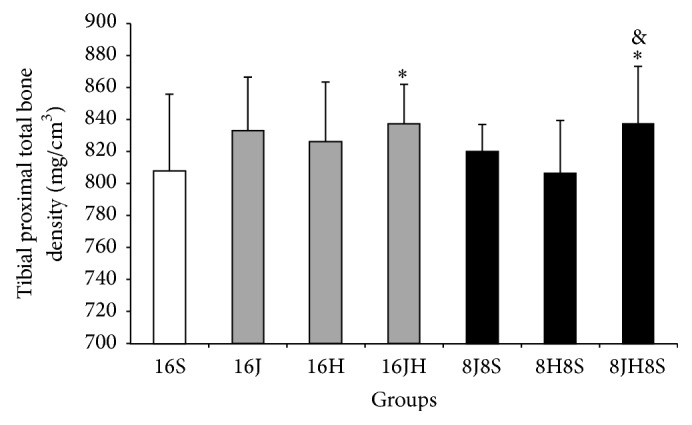
Mean proximal total bone density of the tibiae of the rats (means ± SD). ^*∗*^ *p* < 0.01 as compared to 16S. ^&^ *p* < 0.01 as compared to 8H8S. 8 = 8 weeks, 16 = 16 weeks, S = sedentary without honey supplementation, H = honey supplementation, and J = jumping exercise.

**Figure 2:**
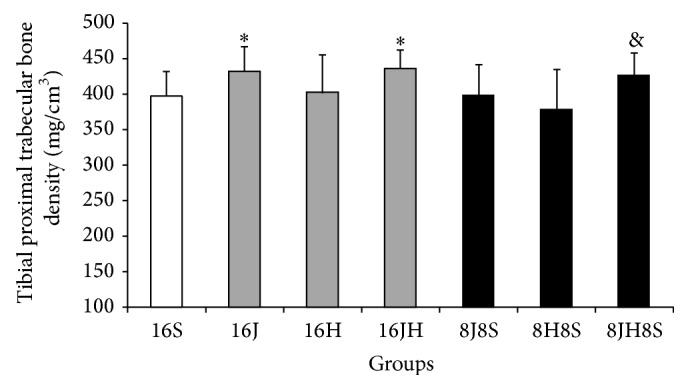
Mean proximal trabecular bone density of the tibiae of the rats (means ± SD). ^*∗*^ *p* < 0.01 as compared to 16S. ^&^ *p* < 0.01 as compared to 8H8S. 8 = 8 weeks, 16 = 16 weeks, S = sedentary without honey supplementation, H = honey supplementation, and J = jumping exercise.

**Figure 3:**
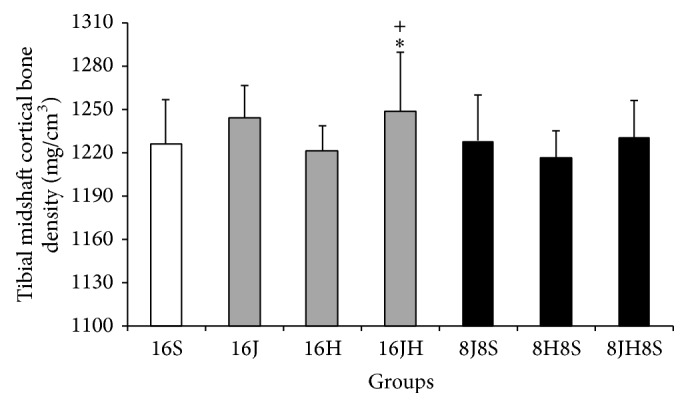
Mean midshaft cortical bone density of the tibiae of the rats (means ± SD). ^*∗*^ *p* < 0.01 as compared to 16S. ^+^ *p* < 0.01 as compared to 16H. 8 = 8 weeks, 16 = 16 weeks, S = sedentary without honey supplementation, H = honey supplementation, and J = jumping exercise.

Mean proximal total bone mineral density, proximal trabecular bone mineral density, and midshaft cortical bone mineral density were significantly (*p* < 0.05) higher in tibiae of the rats in 16 weeks of combined jumping exercise and honey supplementation group (16JH) as compared to control group, that is, 16S (Figures 1, 2, and 3). In addition, 16JH also exhibited significantly (*p* < 0.05) greater midshaft cortical bone mineral density as compared to 16H (Figure 3). Sixteen weeks of continuous jumping exercise (16J) exhibited significantly (*p* < 0.05) greater proximal trabecular bone mineral density when compared with 16S (Figure 2). On the other hand, 16H did not result in any improvement of bone mineral density as compared to 16S (Figures 1, 2, and 3).

After 8 weeks of cessation, 8JH8S showed significantly (*p* < 0.05) greater tibial proximal total bone mineral density than 16S and 8H8S and also greater tibial proximal trabecular bone mineral density as compared to 8H8S (*p* < 0.05). Further statistical analysis revealed that there were no significant differences of bone mineral density parameters in 8JH8S as compared to 16JH (Figures 1, 2, and 3).

### 3.2. Tibial Proximal Cortical Area and Midshaft Cortical Moment of Inertia (MOI)

Results of tibial proximal cortical area and midshaft cortical moment of inertia were illustrated in Table 2. In the present study, tibial cortical area (*p* < 0.05) and midshaft cortical moment of inertia (*p* < 0.01) were significantly higher in 16JH as compared to the control group. 16JH also showed significantly (*p* < 0.05) higher tibial midshaft cortical moment of inertia when compared with 16H (*p* < 0.05). Similarly, 16J also showed greater value as compared to 16S (*p* < 0.05) in both measured parameters.

After the cessation phase, statistical analysis showed that 8JH8S was significantly (*p* < 0.05) higher than 16S in tibial cortical area and midshaft cortical moment of inertia and also had greater (*p* < 0.05) value than 8H8S in cortical area. Additionally, rats in the 8J8S group demonstrated significantly (*p* < 0.05) lower value of cortical area as compared to continuous 16 weeks of jumping exercise. After 8 weeks of cessation, 8H8S exhibited lower tibial cortical area as compared 16H (*p* < 0.05).

### 3.3. Tibial Bone Mechanical Properties

Results of tibial maximum force and tibial energy are shown in Table 3. After the intervention regimen, the rats in the 16 weeks of jumping exercise and honey supplementation group (16JH) had significantly higher tibial maximum force (*p* < 0.01) and tibial energy (*p* < 0.05) compared to the control group. Tibial maximum force was also significantly (*p* < 0.01) higher in 16J as compared to 16S. No significant differences of maximum load were observed after the cessation of exercise and/or honey supplementation in 8J8S, 8H8S, and 8JH8S when compared with the control group, whereas tibial energy was significantly (*p* < 0.05) greater after the cessation of exercise and honey supplementation in 8JH8S as compared to 16S. There were no significant differences in bone mechanical parameters in 16H and 8H8S as compared to 16S, respectively.

## 4. Discussion

The main findings of the present study were that there were overall improvements in bone mineral density measurements, cortical area, midshaft moment of inertia, and mechanical parameters of tibia after 16 weeks of continuous jumping exercise and honey supplementation. Additionally, after 8 weeks of sedentary conditions without supplementation, tibial proximal total bone density, cortical area, midshaft cortical moment of inertia, and energy were significantly higher in 8JH8S as compared to 16S, which indicated that the beneficial effects of 8 weeks of jumping exercise and honey supplementation could still be maintained even after the 8 weeks of cessation of exercise and supplementation.

In the present study, 16 weeks of jumping exercise combined with honey supplementation increased tibial proximal total bone density, proximal trabecular bone density, midshaft cortical bone density, tibial cortical area, midshaft cortical moment of inertia, maximum force, and energy as compared to sedentary control group. However, 16 weeks of jumping exercise alone merely showed improvement in tibial proximal trabecular bone density, cortical area, midshaft cortical moment of inertia, and maximum force. These findings indicated that more beneficial bone effects were observed when jumping exercise was combined with honey supplementation compared with jumping exercise alone.

High impact exercise is considered to be one of the effective exercises on osteogenic response; its intermittent dynamic loading is known to be more effective in increasing bone health than static loading [25]. The dynamic loading of high impact exercise can produce a high magnitude strain and strain rate on bones, and they are important factors for osteogenic response [26]. Jumping exercise was prescribed in the present study, based on the fact that it is a type of high impact weight bearing exercise which can produce large ground reaction force and impose strong muscular contraction force on bone. Jumping exercise is considered more effective as compared to low impact or non-weight-bearing exercises to increase bone mass and strength [27].

The positive effects of exercise on bone as observed in the present study have been reported by several related previous studies. Umemura et al. [25] reported that 3 weeks of daily jumping exercise increased femoral cortical area and total bone mineral density in 4-week-old mice. There was a significant increase in tibial bone mass, strength, and cortical areas in jump-exercised rats [26]. In another study by Umemura et al. [9], it was shown that three sessions per week of ten jumps per session resulted in significantly greater tibia fat-free dry weight, strength, and midshaft cortical area. In general, the findings of the present study along with the previous mentioned studies indicated that the responses to mechanical loading induced by jumping exercise resulted in increases in bone properties in rats.

It was hypothesized by the authors that combined physical activity and supplementation may play a vital role in enhancing and maintenance of bone gain. The present findings of increases in all the measured parameters with 16 weeks of combined jumping exercise and honey supplementation have confirmed our hypothesis. In our previous study, it was found that a shorter duration, that is, 8 weeks of combined jumping exercise and honey supplementation, elicited more discernible beneficial effects on tibia and femur bones when compared to either jumping exercise or honey supplementation alone in young female rats [23, 28].

Honey has been reported to increase intestinal calcium absorption due to its carbohydrate nature [19]. It is speculated that honey supplementation during jumping exercise in the rats may have increased the intestinal calcium absorption and subsequently enhanced bone mineral density and cortical area of the rats. The evidence that the beneficial effect of exercise was enhanced when there was a presence of adequate calcium intake has been reported by Welch and Weaver [29], which demonstrated that a combination of moderate-impact exercise and adequate calcium intake increased bone strength during childhood. Specker and Binkley [30] also reported that, among children receiving calcium, the subjects' bone thickness and area of the leg were larger when combined with physical activity.

In the present study, 8 weeks of jumping exercise and honey supplementation followed by 8 weeks of sedentary condition without supplementation (8JH8S) exhibited significantly greater tibial proximal total bone density, cortical area, midshaft cortical moment of inertia, and energy as compared to control group (16S), indicating that the beneficial effects of jumping exercise and honey supplementation in proximal total bone density and cortical area, midshaft cortical moment of inertia, and energy may still be maintained after 8 weeks of cessation of the intervention.

Equivocal findings were reported with regard to preservation of exercise-induced bone gain after the exercise intervention was terminated. In an animal study, Umemura et al. [9] reported that the effects of jump training on tibial bone strength, mass, and width of the tibial diaphysis were preserved after 24 weeks of detraining. Similarly, Singh et al. [20] and Ooi et al. [31] indicated that high impact jump exercise per session, performed 5 days per week for 4 weeks, can lead to an increased cortical bone with enhanced periosteal bone formation, which was also maintained after cessation of exercise. The authors suggested that high impact jump exercise may provide greater safety margin against disuse-related or/and age-related bone loss and skeletal fragility later in life. It can also be speculated that when improvements in bone mass or strength were associated with increase in cortical area or bone enlargement, the benefits may persevere after detraining.

On the other hand, as reported in the present study, after 8 weeks of cessation of jumping exercise in 8J8S, there were no significant differences in the measured bone parameters in this group of rats as compared to the control rats. This finding is in agreement with the study by Yeh and Aloia [21] on young female rats, who reported that a 4-week period of deconditioning was sufficient to diminish the beneficial effects of 8 weeks of exercise on bone mineral content, bone mineral density, and dynamic parameters of bone formation and resorption. Similarly, Iwamoto et al. [22] reported that, after deconditioning, exercised rats lost the beneficial effects gained through 8 weeks of exercise and their bone parameters were reduced to levels not different from the sedentary control. The plausible explanation for the inconsistencies among the previous studies might be related to the different bone site responses, as well as different loading types and assessment methods. Likewise, differences in age, strain of the animals, or the experimental environment, for example, food given, room temperature, and humidity, could be other possible factors to bone response to mechanical loading. The results of the present study along with the findings of some previous studies indicated that 16 weeks of jumping exercise resulted in increase in tibial proximal trabecular bone density, cortical area, midshaft cortical moment of inertia, and maximum force. However, no beneficial effects were observed after 8 weeks of jumping exercise followed by 8 weeks of sedentary, implying that bone gains through jumping exercise alone were not preserved after the cessation. Therefore, continuous exercise is needed for the maintenance of bone health in female rats.

In the present study, the beneficial effects of jumping exercise were preserved after the cessation only when exercise was combined with honey supplementation. The absorption of calcium contained in the honey into the blood during exercise and subsequent enhancement of bone properties may have played a role in eliciting the positive effects of combined exercise and honey supplementation as observed in the present study. Honey is a commonly used sweetener consisting of the carbohydrates of fructose, glucose, and raffinose. Various studies with animal models have documented the calcium uptake-enhancing effects of raffinose [32, 33] which was also present in honey used for this study.

The mechanism in which honey and other nondigestible carbohydrates can increase calcium absorption as mentioned in Ariefdjohan et al. [19] and Scholz-Ahrens et al. [34] could be that, in the gastrointestinal tract, nondigestible carbohydrates resist hydrolysis by digestive enzymes, so that carbohydrates pass through the small intestine and eventually reach the cecum and colon. At these sites, they are fermented by the residing bacteria to produce byproducts such as short-chain fatty acids, resulting in a decrease in intestinal pH, which leads to an increase in mineral solubility such as calcium. Ariefdjohan et al. [19] also reported that rats fed with 800 mg of honey for 2 days had significantly higher percent of calcium absorption than those fed with glucose-fructose mixture or a low dose of raffinose. This suggests that there may be other factors such as flavonoids, vitamin D, and vitamin K in honey that contribute to its calcium absorption enhancing effect. Flavonoids consist of the most important category of phenolic compounds in honey and have a potential inhibitory effect on bone resorption [35].

Honey supplementation alone for 16 weeks did not significantly improve bone properties in the present study. Similar findings have been reported by Ariefdjohan et al. [19] after chronic long-term feeding of honey. In their study, 5% and 10% of dietary honey for duration of 8 weeks did not improve the calcium absorption, femur bone length, strength, bone mineral content, and bone mineral density compared to the control group. In a human study, Malaysian Tualang honey supplementation in postmenopausal women did not result in any improvement in bone mineral density of femur and lumbar spine as compared to those who received hormone replacement therapy [36]. One of the probable reasons reported by Hussain et al. [36] for the failure to observe any effect of honey on the bone health could be the short duration of the study, which was only four months. On the other hand, low dose of Tualang honey showed more improvement in trabecular bone structure than calcium receiving rats [37]. Inconsistent results between the current study and the study done by Mohamad et al. [37] could be due to the differences in physiological condition of the rats. The animals used in the present study were healthy female rats, whereas in their study, ovariectomized rats under the condition of hormone deficiency were used. In another study by Chepulis and Starkey [38], bone mineral density was observed to be significantly increased in honey-fed rats compared with those fed with sugar-free diet for 52 weeks. Containing no sugar in the diet of control group in Chepulis and Starkey's [38] study could be the reason for differences in their findings and the present study, since the animals' diet in the present study contained carbohydrate. To support the findings of the present study, Chepulis and Starkey [38] also reported no differences in bone mineral density between honey- and sucrose-fed rats.

The lack of long-term nutritional benefit may be attributed to several factors. One of the factors could be that honey contains calcium, and as reported by Brommage et al. [39], upon prolonged administration of a highly bioavailable calcium load, colonic calcium absorption adapts to maintain homeostasis. The parathyroid hormone-vitamin mechanism may be suppressed by the initial increase in calcium absorption. Subsequently, this event downregulates the active transport of calcium absorption. Inconsistent findings were revealed by Chepulis and Starkey [38] where bone mineral density was observed to be significantly increased in honey-fed rats compared with those fed with sugar-free diet for 52 weeks.

In the present study, 8 weeks of honey supplementation followed by 8 weeks of sedentary condition without supplementation did not result in any improvement in bone parameters. Tibial cortical area was significantly lower in 8H8S compared with 16H group, indicating that ceasing the honey supplementation leads to diminished gains in bone parameters. However, there is not enough evidence in the current literature to substantiate the effects of cessation of honey supplementation. Nevertheless, it has been reported that withdrawal from other supplementation such as calcium reduced the bone mineral content acquisition to the level that was not significantly different from control group after 1 year of follow-up in 7-year-old children, suggesting that bone acquisition rate was greater during the administration of calcium supplement and this rate deteriorated when calcium supplementation was withdrawn [40]. The authors speculated that calcium supplements given to growing children may slow down bone turnover rate. A reduction in bone turnover allows more mineralization to take place in the bone remodeling units, leading to a decline in the skeletal calcium space in the bone matrix. As a result, an apparent increase in bone mineral density can be detected by photon absorptiometry. After the calcium load was removed, the rate of the bone remodeling process may reverse [40].

Exercise and nutrition are both independently important for skeletal development, though it is still not clear whether combination of these factors interacts to enhance skeletal health during growth. Current evidence suggests that adequate intake of dietary calcium, vitamin D, and protein is accepted as important to optimize bone health during growth; however, exercise would appear to be more important for optimizing bone strength because it has a direct effect via loading on bone mass and structural properties, whereas nutritional factors appear to have an indirect effect through hormonal factors on bone mass [41]. To date, to our knowledge no studies on effects of cessation with exercise combined with nutritional supplementation have involved as many experimental groups as in the present study. The limitations of the present study were that blood vitamin D, calcium and phosphorus, and blood bone metabolism markers concentrations were not measured, and it is suggested to include these measured parameters in further studies. Additionally, inclusion of histological analysis is warranted in future studies.

In conclusion, the present study found that continuous 16 weeks of jumping exercise and honey supplementation elicited more beneficial effects on tibia bone in comparison with jumping exercise or honey supplementation alone. Additionally, the beneficial effects of 8 weeks of jumping exercise and honey supplementation on tibia bone properties could still be maintained even after 8 weeks of cessation of exercise and supplementation. Therefore, the results obtained from the present study can be used as a foundation for studies involving human subjects, while determining the effectiveness of combined jumping exercises and honey supplementation as guidelines for enhancement of bone health in humans.

## Figures and Tables

**Table 1:** Initial and final body weight of the rats (mean ± SD).

Groups	Initial body weight (g)	Final body weight (g)
16S	200.3 ± 7.6	248.1 ± 15.7
16J	202.9 ± 14.1	259.9 ± 28.3
16H	200.1 ± 12.6	246.1 ± 22.3
16JH	203.9 ± 12.9	252.0 ± 24.0
8J8S	203.3 ± 10.0	246.6 ± 15.0
8H8S	203.2 ± 11.6	251.5 ± 14.5
8JH8S	202.1 ± 10.7	247.7 ± 9.5

8 = 8 weeks, 16 = 16 weeks, S = sedentary without honey supplementation, H = honey supplementation, and J = jumping exercise.

**Table 2:** Cortical area and midshaft cortical moment of inertia of the tibiae of the rats (mean ± SD).

Groups	Tibial cortical area (mm^2^)	Tibial midshaft cortical moment of inertia (kg/m^2^)
16S	8.1 ± 0.9	3.41 ± 0.65
16J	9.1 ± 0.9^*∗*^	3.99 ± 0.79^*∗*^
16H	8.2 ± 1.2	3.53 ± 0.44
16JH	9.1 ± 0.8^*∗*^	4.07 ± 0.52^*∗∗*,+^
8J8S	8.2 ± 0.6^#^	3.77 ± 0.76
8H8S	7.9 ± 1.3^+^	3.49 ± 0.58
8JH8S	8.9 ± 1.2^*∗*,&^	3.93 ± 0.35^*∗*^

^*∗*^ *p* < 0.05; ^*∗∗*^ *p* < 0.01 as compared to 16S.

^&^ *p* < 0.05 as compared to 8H8S.

^+^ *p* < 0.05 as compared to 16H.

^#^ *p* < 0.05 as compared to 16J.

8 = 8 weeks, 16 = 16 weeks, S = sedentary without honey supplementation, H = honey supplementation, and J = jumping exercise.

**Table 3:** Maximum force and energy of the tibiae of the rats (mean ± SD).

Groups	Tibial maximum force (N)	Tibial energy (kg/m^2^)
16S	79.30 ± 6.77	39.57 ± 7.05
16J	87.09 ± 6.90^*∗∗*^	46.73 ± 5.31
16H	81.38 ± 8.87	45.75 ± 8.07
16JH	86.91 ± 6.56^*∗∗*^	47.44 ± 6.53^*∗*^
8J8S	83.12 ± 6.20	43.77 ± 3.93
8H8S	79.59 ± 6.24	40.69 ± 4.00
8JH8S	83.70 ± 4.75	47.92 ± 9.21^*∗*^

^*∗*^ *p* < 0.05; ^*∗∗*^ *p* < 0.01 as compared to 16S.

8 = 8 weeks, 16 = 16 weeks, S = sedentary without honey supplementation, H = honey supplementation, and J = jumping exercise.
